# Impact of Falls and Fear of Falling on Participation, Autonomy and Life Satisfaction in the First Year After Spinal Cord Injury

**DOI:** 10.3389/fresc.2022.903097

**Published:** 2022-06-09

**Authors:** Katherine Chan, Olinda Habib Perez, Hardeep Singh, Andresa R. Marinho-Buzelli, Sander L. Hitzig, Kristin E. Musselman

**Affiliations:** ^1^KITE-Toronto Rehabilitation Institute, University Health Network, Toronto, ON, Canada; ^2^Department of Occupational Science and Occupational Therapy, Temerty Faculty of Medicine, University of Toronto, Toronto, ON, Canada; ^3^Rehabilitation Sciences Institute, Temerty Faculty of Medicine, University of Toronto, Toronto, ON, Canada; ^4^St. John's Rehab Research Program, Sunnybrook Health Sciences Centre, Toronto, ON, Canada; ^5^Department of Physical Therapy, Temerty Faculty of Medicine, University of Toronto, Toronto, ON, Canada

**Keywords:** spinal cord injury, falls, fear of falling, participation, autonomy, life satisfaction

## Abstract

**Introduction:**

Individuals with spinal cord injury (SCI) experience reduced participation in meaningful activities, leading to reduced social engagement and negative psychological impact. Two factors that may affect participation post-SCI are fall status (e.g., having experienced a fall) and having a fear of falling. Our objective was to examine if and how fall status and fear of falling impact participation, autonomy and life satisfaction in the first year post-injury.

**Methods:**

Adult inpatients of a SCI rehabilitation hospital were recruited. Following discharge, falls were tracked for 6 months and participants who fell at least once were categorized as “fallers”. At the end of the 6-month period, the Impact on Participation and Autonomy Questionnaire and Life Satisfaction Questionnaire 9 were administered, and participants were asked if they had a fear of falling (i.e., an ongoing concern about falling leading to the avoidance of activities they are capable of doing). Falls were reported using descriptive statistics. Ordinary least squares regression was used to evaluate the relationships between the independent variables (i.e., fall status and fear of falling) and each dependent variable (i.e., questionnaire scores).

**Results:**

Seventy-one individuals were enrolled in the study; however, 11 participants were lost to follow-up. The included participants (*n* = 60) were 58.4 ± 14.6 years old and 99 ± 60.3 days post-injury. Over one third (38.3%) of participants fell over the 6-month tracking period. Twenty-seven participants (45%) reported a fear of falling and 14 (51.9%) of these participants were fallers. Fear of falling significantly predicted scores of autonomy indoors (β = 3.38, *p* = 0.04), autonomy outdoors (β = 2.62, *p* = 0.04) and family role (β = 3.52, *p* = 0.05).

**Conclusion:**

Individuals with subacute SCI and a fear of falling experienced reduced participation and autonomy, but with no differences in life satisfaction compared to those without a fear of falling. In contrast, having experienced a fall did not impact participation, autonomy or life satisfaction. In the first year after SCI, rehabilitation programs should place specific attention on the presence of fear of falling to help individuals with SCI prepare for everyday mobility challenges.

## Introduction

Spinal cord injury (SCI) is a significant, life-altering event. Damage to the spinal cord may be caused by a sudden traumatic incident, such as a motor vehicle accident, or a non-traumatic etiology, such as a spinal tumor or vascular abnormality. In both cases, spinal cord damage causes sensory, motor and autonomic dysfunctions that impact independence and participation in daily life. Following a SCI, individuals often participate in intensive rehabilitation to optimize their ability to move and self-care, as well as facilitate independent living, community integration and participation in meaningful activities.

The World Health Organization defines participation as “the involvement in a life situation” (1), and encompasses a wide range of tasks and skills. These include communication, mobility, self-care, interpersonal interactions and relationships, and engagement in domestic, community, social and civic life (1, 2). Previous studies have reported a less than optimal level of participation in individuals with SCI (3, 4). More than 50% of community-dwelling individuals living with SCI report one or more significant challenges with their participation (3), as well as restrictions in autonomy (5). Changes in the type of activities and roles pursued post-SCI have been reported, with more time spent on self-care and less time on work or study (4, 6).

Since greater participation is associated with greater quality of life after SCI (7), there is significant motivation to understand the factors influencing participation after sustaining a SCI. Older age at time of SCI, cognitive deficits, more significant medical complications, and reduced social support are linked to lower participation levels (3, 5, 8). Two factors that may influence participation post-SCI, but have received little study to date (9), are having experienced a fall [i.e., coming to rest inadvertently on the ground or other lower level (1)] and having a fear of falling (i.e., a lasting concern about falling that causes an individual to avoid or curtail activities that they are capable of performing) (10). Among older adults, both a history of multiple previous falls and a fear of falling independently predict restrictions in daily activities, such as mobilizing outdoors and performing self-care and household activities (11). Previous falls may cause physical injury that impedes function and/or lower self-efficacy related to avoiding falls (i.e., falls self-efficacy), both of which limit participation in daily activities (11). Similarly, a fear of falling, irrespective of fall history, may cause self-induced participation restrictions (12). This consequence of a fear of falling is often explained using a self-efficacy framework (13), in which low falls self-efficacy is a mediator between a fear of falling and behavior, such as restricting participation in activities and roles (12).

Previous falls and fear of falling are relevant factors to consider when studying participation after SCI. Falling is common among those living with chronic SCI (i.e., >1 year post-injury); every year, 69–78% of individuals with chronic SCI fall at least once (14). Similarly, 50–73% of individuals with chronic SCI report having a fear of falling (9, 15–18). To date, one study investigated the impact of falls and fear of falling on standardized questionnaires of participation and quality of life in 65 individuals with chronic SCI (9). Interestingly, the findings suggested decreased participation and quality of life were associated with a fear of falling, but not experiencing falls (9).

Compared with the chronic phase of SCI, falls are less common early after a SCI while receiving inpatient hospital care; 7–20% of inpatients with SCI have been reported to fall (19–21). Individuals with subacute SCI are likely at a greater risk of falling once discharged home; however, little is known about the incidence of falls and fear of falling, and their impact on participation in the subacute phase of SCI recovery. A recent qualitative study explored the impact of falls and fall risk on community-dwelling individuals who had been living with a SCI for <1 year (22). Participants reported that they associated their fall risk with decreased independence, quality of life, and confidence, as well as increased negative emotions, such as fear and anxiety (22). Further investigation using quantitative methods is needed to measure the impact of falling and fear of falling on participation early after a SCI, and to compare the findings with prior qualitative research (22).

As the subacute phase of SCI is typically a time of intense rehabilitation, understanding the relationships between falls, fear of falling and participation may inform the content of impactful fall prevention initiatives. Hence, our objective was to determine the impact of experiencing a fall and having a fear of falling on participation, autonomy and quality of life in individuals with subacute SCI. Based on the findings of the prior qualitative research (22), we hypothesized that both a fear of falling and falling would be associated with lower scores on measures of participation, autonomy and quality of life.

## Materials and Methods

This longitudinal study was part of a larger mixed methods project that investigated the causes and consequences of falls across the continuum of care (i.e., from inpatient rehabilitation to community living) in Canadians with subacute SCI. Ethical approval was received from the Research Ethics Board of the University Health Network.

### Participants

Eligible participants were identified by the Central Recruitment service at the Lyndhurst Centre, Toronto Rehabilitation Institute, University Health Network (23). Participants were approached by a research team member to obtain written, informed consent. Eligible participants met the following criteria: (1) were at least 18 years old; (2) had a traumatic or non-traumatic and non-progressive cause of SCI; (3) had an AIS rating of A-D (24); (4) were an inpatient at the Lyndhurst Centre at the time of enrollment; and (5) did not have other significant co-morbidities that affected their balance (e.g., stroke).

A target sample size of 64 was calculated (25) using data from a previous study involving individuals with SCI and the Impact on Participation and Autonomy Questionnaire (IPA) (26), which was the measure of participation and autonomy used in the present study. An alpha value of 0.05, a beta value of 0.10, and a 45% fall rate for the sample (27) were used in the sample size calculation. We aimed to recruit 71 individuals to allow for 10% attrition.

### Data Collection

One week prior to discharge from inpatient rehabilitation, demographic information (e.g., age, sex), injury-related information (e.g., time post-injury, mechanism of injury) and mobility status (i.e. uses a wheelchair or ambulates) were collected from participants. Individuals who used a wheelchair (manual or power) for at least 4 h per day were considered wheelchair users (28). AIS rating and other injury-related information that could not be provided by the participants were extracted from their medical charts with their consent. At this time, participants were instructed to document each fall they experienced during the 6 months following discharge. Participants were given the World Health Organization's definition of a fall for reference: “coming to rest inadvertently on the ground or other lower level” (1). Falls were documented in an online survey (Qualtrics Survey Software) or on paper, according to the preference of each participant. Participants were asked to complete the survey within 24 h of experiencing the fall to reduce recall bias. A research team member called participants every 3–4 weeks to ensure that the fall surveys were being completed. For participants who documented the fall on paper, a research team member entered this information into the Qualtrics Survey platform during the phone call. We have used these methods to track falls in prior research (29–31).

At the end of the 6 months, a research team member administered two questionnaires: the IPA and the Life Satisfaction Questionnaire 9 (LiSAT-9) (described below). These questionnaires were conducted in person or over the phone. Each participant's current mobility status was recorded. Participants were also asked if they had a fear of falling; more specifically, they were asked, “Do you have a fear of falling, defined as “a lasting concern about falling causing you to avoid or curtail activities you felt you were capable of doing?” (10).

### Impact on Participation and Autonomy Questionnaire (IPA)

The IPA was administered to quantify the impact of SCI on participants' participation and autonomy. For each question, participants' responses were guided by a 5-point Likert scale with the following options: “0-very good”, “1-good”, “2-fair”, “3-poor” or “4-very poor”. Responses were summed into five subscale categories: autonomy indoors, autonomy outdoors, family roles, social relations, and paid work and education (26, 32). A higher score indicated a greater negative impact on participation and autonomy. The IPA has excellent test-retest reliability (26) and has demonstrated content validity (26) in the SCI population.

### Life Satisfaction Questionnaire 9 (LiSAT-9)

The LiSAT-9 was used to measure life satisfaction, which refers to an “individuals' assessment of their emotions, happiness, or satisfaction with respect to their expectation and achievements” (33). The LiSAT-9 includes nine items that assess total life satisfaction, as well as eight domains, such as occupation/employment, management of self-care, leisure activities, and relationships with partners and friends (34). Participants rated how satisfied they were with each domain on a 6-point Likert scale (1 = very dissatisfying to 6 = very satisfying); hence, a higher score indicated better life satisfaction in that domain. The LiSAT-9 total score was calculated by averaging the scores from the nine items. The LiSAT-9 is a valid and responsive measure for the SCI population (33, 34).

### Data Analysis

Demographic information, injury-related data and mobility status were reported as mean ±1 standard deviation (SD) or count (percentage), as appropriate. These data were reported for the entire sample and according to fall status and fear of falling status. Participants were classified as “fallers” if they experienced one or more falls in the 6 months since hospital discharge or “non-fallers” if they did not experience any falls. The percentage of the sample with one or more falls was reported, as was the percentage of the sample with a fear of falling. Independent *t*-tests and chi-square tests were used to compare interval-level variables (e.g., age) and categorical variables (e.g., sex), respectively, between participants with differing fall statuses, as well as between participants who did and did not report a fear of falling. Spearman's correlation was used to examine the relationship between fall status and fear of falling status.

The IPA subscale scores and LiSAT-9 total score were calculated for each participant following the instruments' instructions. The paid work and education subscale of the IPA was excluded from the analyses as numerous participants responded to the questions in this section with “not applicable” (i.e., not enrolled in school, not working or retired). Scores on questionnaires were reported according to fall status and fear of falling status. Questionnaire scores were reported as median (interquartile range, IQR), with the exception of LiSAT-9 total score, which was reported as mean (±1 SD). Ordinary least squares regression was used to examine the impact of the independent variables (i.e., fall status and fear of falling status) on each dependent variable separately (i.e., IPA subscale scores and the LiSAT-9 total score). As an additional exploratory analysis, we conducted ordinary least squares regression with four independent variables (i.e., non-faller without a fear of falling, non-faller with a fear of falling, faller without a fear of falling, and faller with a fear of falling). The regressions were completed in the statmodels module of Python (35). All other statistical tests were conducted on IBM SPSS Statistics 27. Alpha was set to 0.05.

## Results

A total of 71 individuals consented to participate in the study. Eleven participants were lost to follow-up; therefore, 60 participants (13 female and 47 male) were included for the analyses. Participants were 58.4 ± 14.6 years old and 99 ± 60.3 days post-injury. Just under half of the participants (48.3%) had a traumatic cause of injury. A majority (73.4%) had a motor incomplete SCI (i.e., American Spinal Injury Association Impairment Scale grade of C or D). Thirty participants (50%) ambulated while 20 participants (33.3%) used a wheelchair for mobility. The remaining 10 participants (16.7%) were using a wheelchair at the time of discharge from inpatient rehabilitation, but were ambulators at 6 months post-discharge. Participant demographics are reported in Table 1.

**Table 1:** Participant demographics.

	**All participants (*n =* 60)**	**Fallers (*n =* 23)**	**Non-fallers (*n =* 37)**	***p*-value**	**Participants with FOF** **(*n =* 27)**	**Participants with no FOF (*n =* 33)**	***p*-value**
Sex,
F	13 (22%)	3 (13.0%)	10 (27.0%)	0.56	4 (14.8%)	9 (27.3%)	0.24
M	47 (78%)	20 (87.0%)	27 (73.0%)		23 (85.2%)	24 (72.7%)	
Age, years	58.8 ± 14.6	53.5 ± 16.0	61.5 ± 13.0	0.04^*^	58.9 ± 12.5	58.1 ± 16.3	0.84
Time post-injury, days	99.0 ± 60.3	86.4 ± 39.6	106.9 ± 69.6	0.21	98.0 ± 49	99.8 ± 69.0	0.91
Level of injury							
Paraplegia	28 (46.7%)	13 (56.5%)	15 (40.5%)	0.19	11 (40.7%)	17 (51.5%)	0.42
Tetraplegia	32 (53.3%)	10 (43.5%)	22 (59.5%)		16 (59.3%)	16 (48.5%)	
Mechanism of injury							
Traumatic	29 (48.3%)	11 (47.8%)	18 (48.6%)	0.43	13 (48.1%)	16 (48.5%)	0.98
Non-traumatic	31 (51.7%)	12 (52.2%)	19 (51.4%)		14 (51.9%)	17 (51.5%)	
Mobility status							
Ambulates	30 (50.0%)	11 (47.8%)	19 (51.4%)		14 (51.9%)	16 (48.5%)	
Uses wheelchair	20 (33.3%)	6 (26.1%)	14 (37.8%)	0.43	7 (25.9%)	13 (39.4%)	0.55
Wheelchair → Ambulates^∧^	10 (16.7%)	6 (26.1%)	4 (10.8%)		6 (22.2%)	4 (12.1%)	
Severity of injury							
Motor Complete	12 (44.0%)	5 (21.7%)	7 (18.9%)	0.64	4 (14.8%)	8 (24.2%)	0.73
Motor Incomplete	44 (73.3%)	17 (73.9%)	27 (73.0%)		21 (77.8%)	23 (69.7%)	
AIS not completed	4 (6.7%)	1 (4.3%)	3 (8.1%)	-	2 (7.4%)	2 (6.0%)	-

*Values are reported as mean ± 1 standard deviation or count (%), as appropriate*.

∧ *Used a wheelchair at discharge from inpatient rehabilitation, but transitioned to an ambulator 6 months post-discharge*.

* *Denotes statistically significant p-value. FOF, fear of falling; AIS, American Spinal Injury Association Impairment Scale*.

More than one third (38.3%) of participants experienced at least one fall over the 6-month tracking period and were classified as fallers. Two participants experienced a fall that required medical attention (i.e., fractured toe, concussion). Fallers were significantly younger than non-fallers (*p* = 0.04), but no other significant differences in demographic information, injury-related characteristics or mobility status were found between fallers and non-fallers. Twenty-seven participants (45%) reported a fear of falling, with 14 (51.9%) of these individuals experiencing one or more falls over the tracking period. There were no differences in demographic information, injury-related characteristics or mobility status between those who did and did not report a fear of falling. No relationship was found between fall status and fear of falling status (ρ = −0.24, *p* = 0.86).

Scores on the IPA and LiSAT-9 are reported according to fall status and fear of falling status separately (Table 2). The overall regression models with two independent variables (i.e., fall status and fear of falling status) were not statistically significant (*R*^2^ = 0.03–0.08, F_2, 57_ = 0.92–2.55, *p* = 0.08–0.18 for IPA subscale scores and LiSAT-9 total score). However, fear of falling status significantly predicted scores on the autonomy indoors (β = 3.38, *p* = 0.04), autonomy outdoors (β = 2.62, *p* = 0.04) and family role (β = 3.52, *p* = 0.05) subscales of the IPA (Table 3), such that reporting a fear of falling was associated with higher scores (i.e., less participation and autonomy).

**Table 2:** IPA and LiSAT-9 scores.

						**Exploratory analysis**			
		**Falling status**		**Fear of falling status**		**Non-faller and No FOF** **(*n =* 20)**	**Non-faller and FOF (*n =* 17)**	**Faller and No FOF** **(*n =* 13)**	**Faller and FOF** **(*n =* 10)**
		**Faller**	**Non-faller**	**Yes**	**No**				
	IPA - Autonomy Indoors	3 (7)	5 (10)	8 (10)	2 (7)	2 (8.5)	8 (7)	2 (5)	6.5 (12.5)
	IPA - Family Role	8 (9)	7 (11)	9 (12)	6 (11)	6.5 (10.5)	9 (10)	6 (9)	10 (11.75)
	IPA - Autonomy Outdoors	7 (6)	7 (8)	8 (7.5)	6 (6)	6 (6.5)	8 (8)	6 (4)	8.5 (7.25)
	IPA - Social Life and Relationships	6 (5)	3 (8)	6 (8.5)	4 (6)	3 (7.5)	5 (8)	6 (5)	6.5 (7.25)
	LiSAT-9 Total	4.2 ± 1.1	4.4 ± 1.0	4.2 ± 1.2	4.5 ± 0.9	4.5 ± 1.0	4.3 ± 1.1	4.4 ± 0.8	3.9 ± 1.4

*Values are reported as median (IQR) or mean ± 1 standard deviation, as appropriate. FOF, fear of falling; IPA, Impact on Participation and Autonomy Questionnaire; LiSAT-9, Life Satisfaction Questionnaire-9*.

**Table 3:** Ordinary least squares regression.

					**Exploratory analysis**							
		**Falling status** **(*****n** **=*** **23)**		**Fear of falling status (*****n** **=*** **37)**		**Non-faller and No FOF (*****n** **=*** **20)**		**Non-faller and FOF (*****n** **=*** **17)**		**Faller and No FOF (*****n** **=*** **13)**		**Faller and FOF** **(*****n** **=*** **10)**	
		**β**	***p*-value**	**β**	***p*-value**	**β**	***p*-value**	**β**	***p*-value**	**β**	***p*-value**	**β**	***p*-value**
	IPA - Autonomy Indoors	−1.27	0.44	3.38	0.04*	0.66	0.60	2.88	0.04*	−1.96	0.19	3.31	0.05*
	IPA - Family Role	1.12	0.54	3.52	0.05*	−0.08	0.95	2.32	0.12	−0.27	0.87	5.07	0.01*
	IPA - Autonomy Outdoors	0.52	0.68	2.62	0.04*	0.10	0.91	2.37	0.02*	0.21	0.85	3.40	0.01*
	IPA - Social Life and Relationships	0.96	0.53	2.60	0.08	−0.15	0.90	1.61	0.20	−0.17	0.90	3.80	0.02*
	LiSAT-9 Total	−0.23	0.41	−0.30	0.28	1.06	<0.01*	0.91	<0.01*	1.00	<0.01*	0.47	0.10

^*^denotes significant p-values.

*FOF, fear of falling; IPA, Impact on Autonomy and Participation Questionnaire; LiSAT-9, Life Satisfaction Questionnaire-9*.

When the regression models were run with four independent variables (i.e., non-faller without a fear of falling, non-faller with a fear of falling, faller without a fear of falling, and faller with a fear of falling), the overall models were not statistically significant (*R*^2^ = 0.04–0.10, F_4, 55_ = 0.76–1.98, *p* = 0.13–0.52 for IPA subscale scores and LiSAT-9 total score). However, being a faller with a fear of falling was a statistically significant predictor of higher scores on the IPA subscales (i.e., lower participation and autonomy): autonomy indoors (β = 3.31, *p* = 0.05), family role (β = 5.07, *p* = 0.01), autonomy outdoors (β = 3.40, *p* = 0.01) and social life and relationships (β = 3.80, *p* = 0.02). Being a non-faller with a fear of falling was also a significant predictor of higher scores on two IPA subscales: autonomy indoors (β = 2.88, *p* = 0.04) and autonomy outdoors (β = 2.37, *p* = 0.02). Higher life satisfaction was predicted by being a non-faller (with or without a fear of falling) or a faller without a fear of falling (β = 0.91–1.06, *p* <0.01).

## Discussion

Through this prospective longitudinal study we gained insight into the prevalence of falls and fear of falling during the subacute phase of SCI, as individuals transition from inpatient rehabilitation to community living. Twenty-three (38%) of our participants fell over the first 6 months post-hospital discharge and 27 (45%) reported a fear of falling. We also learned that within the first year after experiencing a SCI, fear of falling status predicted scores of participation in the domains of autonomy indoors, autonomy outdoors and family role. Participants without a fear of falling reported greater participation and autonomy in activities, such as getting around their house, visiting relatives and friends, using leisure time as they choose and completing housework. The majority of questions included in the autonomy indoors/outdoors and family role domains of the IPA queried physical tasks, which may be perceived as having a greater fall risk than the activities queried in the social life and relationships domain (e.g., talking with people and receiving respect). In the exploratory analysis, however, having a fear of falling and having experienced a fall had a more widespread, negative impact on participation, such that these participants had poorer scores in the domain of social life and relationships as well. Moreover, higher life satisfaction was reported in non-fallers with or without a fear of falling and fallers without a fear of falling.

Based on the findings of our recent qualitative study (22), we hypothesized that both a fear of falling and falling would be associated with lower scores on measures of participation, autonomy and quality of life. This hypothesis was not met, as experiencing a fall did not independently predict lower scores on these outcomes. The difference in findings could be explained by the research methods used. It may be difficult to dissociate the impact of falls, living with a high fall risk, and having a fear of falling using qualitative research methods (i.e., semi-structured interviews). In the present study we used standardized questionnaires that have been developed to measure specific constructs, which may better enable us to dissociate the effects of falls from the effects of fear of falling. The findings presented here are more similar to prior quantitative research in community-dwelling individuals with chronic SCI (i.e., 18.89 ± 15.43 years post injury) that reported fear of falling status, not fall status, had an impact on participation (9).

In our study, participants who experienced a fall were found to be significantly younger than non-fallers; however, the difference in mean age between these two groups was small (i.e. 8 years). It is possible that younger individuals are more active during their daily lives (e.g. more likely to be working, parenting, etc.), and this increased activity level may lead to more opportunities to fall. Indeed, higher physical activity levels have been linked to increased risk of falling in individuals with SCI (36). The mean age of all participants in our study was 58.8 ± 14.6 years, which reflects the changing demographics of SCI in Canada. As the incidence of non-traumatic causes of SCI is rising, most Canadians with SCI who are receiving inpatient rehabilitation are >50 years old (37). In addition, the prevalence of falling peaks during middle age (i.e. fourth through sixth decades) for those living with SCI (36, 38), which coincides with the age range of many participants in our study.

We did not find an association between fall status and fear of falling status, which is a finding that has been reported in other clinical populations, such as individuals in the subacute phase of recovery following a stroke (39) and individuals with multiple sclerosis (40). Fear of falling may develop in individuals who have not experienced a fall, a phenomenon reinforced by the findings of our qualitative study (22). Not all participants had experienced a fall since sustaining their SCI, but their perception of their fall risk and their concern about falling was influenced by falls they experienced prior to sustaining their SCI and/or serious falls experienced by family members or friends (22).

Fear of falling is known to impact the participation and life satisfaction of other populations. With respect to participation, prior research demonstrated that in older adults, both with and without a prior history of falls, fear of falling was linked to restrictions in participation (41–44). Similarly, in people living with chronic stroke, those who reported a fear of falling demonstrated significantly reduced activity and participation (45). With respect to life satisfaction, a recent systematic review examining the relationships between falls, fear of falling and quality of life in older, ambulatory adults concluded that both falls and fear of falling were independent predictors of quality of life (46). Sung and colleagues (47) looked at differences in community participation and quality of life in 85 individuals who used a wheelchair full-time, 37 (44%) of whom had a diagnosis of SCI. Participants who reported having a fear of falling demonstrated significantly lower participation and quality of life scores than those who did not report a fear of falling. However, the experience of a fall was not simultaneously studied. It is interesting how our findings suggest less of an effect, if any, of falls and fear of falling on life satisfaction in individuals with subacute SCI. One possible explanation is that individuals with incomplete SCI have expressed a willingness to increase their fall risk in order to maintain an identity considered “normal” (16). Additionally, individuals with subacute SCI may not have had enough time to realize the complete psychosocial impact of falls and fear of falling on life satisfaction compared to their chronic counterparts (9).

The findings of this study support prior work that highlights the importance of addressing fear of falling as a health care concern independently of fall history (48). In addition to negatively impacting participation, as demonstrated here, a fear of falling is also associated with an increased likelihood of experiencing recurrent falls and injurious falls (38). It has been suggested that clinicians should consider two possible scenarios where patients may be restricting participation due to a fear of falling. First, participation in daily activities may be restricted due to a fear of falling, even though the individual has the ability to safely participate. This is a maladaptive behavior that can lead to an increased risk of falls and functional decline (48). In this scenario, interventions that reduce anxiety and increase a sense of control or self-efficacy may be beneficial (48). Second, participation restriction may stem from a fear of falling in someone who accurately perceives their balance impairments, which may be considered an appropriate response. In this case, interventions to address their balance deficits are warranted to prevent participation restriction from becoming a long-term strategy (48). Determining which scenario most closely reflects an individual's motivation for restricting participation is therefore important for intervention planning. As demonstrated in the current study, a sizeable proportion of individuals with subacute SCI, including both individuals who use a wheelchair and individuals who ambulate, have a fear of falling. Hence, addressing this fear early after SCI (i.e., during inpatient and outpatient rehabilitation), as individuals prepare for community integration, is warranted. The majority of fall prevention interventions are delivered while individuals with SCI are receiving inpatient or outpatient care (49, 50); however, the extent to which these interventions focus on a fear of falling is unknown. Moreover, there is a paucity of research on the development and evaluation of interventions that target fear of falling in neurorehabilitation (51), highlighting an area in need of further research.

A comprehensive assessment of fall risk and fear of falling at the end of an individual's inpatient hospital stay is warranted. This assessment should consider the current mobility status of the individual, as fall risk and fall experiences differ between individuals with SCI who ambulate compared to those who use a wheelchair (52, 53). Moreover, it would be important to consider whether an individual's mobility status is likely to change after hospital discharge. For example, 17% of our participants transitioned from a wheelchair to walking during the first 6 months following inpatient discharge. Preparing individuals for this transition in mobility status and the associated changes in fall risk may better prepare individuals with SCI for community integration.

There are two study limitations to note. First, we did not include measures of depression or anxiety in this study, which is a notable limitation. Depression and anxiety were previously found to be associated with a fear of falling in individuals with SCI who use a wheelchair (54). Second, 11 (15%) of our participants were lost to follow-up, which was more than the 10% we estimated. The first 6 months following hospital discharge is likely a busy time for people with SCI as they continue with outpatient rehabilitation and adjust to life with SCI; hence a higher anticipated dropout rate would have been reasonable.

In conclusion, within the first year post-SCI, individuals with a fear of falling reported reduced participation and autonomy in the domains of autonomy indoors/outdoors and family role in comparison to individuals without a fear of falling. Furthermore, fallers with a fear of falling had reduced participation and autonomy not only in the domains of autonomy indoors/outdoors and family role, but also in the domain of social life and relationships, in comparison to non-fallers with a fear of falling. Lastly, fallers and non-fallers without a fear of falling and non-fallers with a fear of falling reported higher life satisfaction than fallers with a fear of falling. Interventions addressing fear of falling may be worthwhile additions to the rehabilitation of individuals with SCI during the subacute phase of recovery.

## Data Availability Statement

The raw data supporting the conclusions of this article will be made available by the authors, without undue reservation.

## Ethics Statement

The studies involving human participants were reviewed and approved by University Health Network Research Ethics Board. The patients/participants provided their written informed consent to participate in this study.

## Author Contributions

KM, HS, and SH contributed to conception and design of the study. KC, OH, and AM-B performed data collection. KC performed data analysis and wrote the first draft of the manuscript. KM wrote sections of the manuscript. All authors contributed to manuscript revision and approved the submitted version.

## Funding

Financial support was provided by the Canadian Institutes of Health Research (Grant CIHR PJT 153017) to KM.

## Conflict of Interest

The authors declare that the research was conducted in the absence of any commercial or financial relationships that could be construed as a potential conflict of interest.

## Publisher's Note

All claims expressed in this article are solely those of the authors and do not necessarily represent those of their affiliated organizations, or those of the publisher, the editors and the reviewers. Any product that may be evaluated in this article, or claim that may be made by its manufacturer, is not guaranteed or endorsed by the publisher.
